# Research on the Impact of Deep Eutectic Solvent and Hot-Water Extraction Methods on the Structure of *Polygonatum sibiricum* Polysaccharides

**DOI:** 10.3390/molecules28196981

**Published:** 2023-10-08

**Authors:** Chunyan Zhang, Lanfang Tang, Xiaojun Su, Qingming Li, Hongying Guo, Zhiwei Liu, Zhongshan Wei, Feng Wang

**Affiliations:** 1 College of Food Science and Technology, Hunan Agricultural University, Changsha 410128, China; zhangchy95@hunau.edu.cn (C.Z.); tanglf2309@163.com (L.T.); suxiaojun5606@163.com (X.S.); lqm@hunau.edu.cn (Q.L.); lily-ghy@163.com (H.G.); zwliu@hunau.edu.cn (Z.L.); 2 College of Landscape Architecture and Art Design, Hunan Agricultural University, Changsha 410128, China; weizs2008@163.com

**Keywords:** *Polygonatum sibiricum* polysaccharides, extraction, purification, structural characterization

## Abstract

Deep eutectic solvent (DES) and hot-water extraction (HWE) methods were utilized to extract polysaccharides from *Polygonatum sibiricum*, referred to as DPsP and WPsP, respectively. The extracted polysaccharides were purified using the Superdex-200 dextran gel purification system, resulting in three components for each type of polysaccharide. The structures of these components were characterized. The molecular weight analysis revealed that DPsP components had slightly larger molecular weights compared with WPsP, with DPsP-A showing a slightly higher dispersity index and broader molecular weight distribution. The main monosaccharide components of both DPsP and WPsP were mannose and glucose, while DPsP exhibited a slightly greater variety of sugar components compared with WPsP. FTIR analysis demonstrated characteristic polysaccharide absorption peaks in all six PSP components, with a predominance of acidic pyranose sugars. NMR analysis revealed the presence of pyranose sugars, including rhamnose and sugar aldehyde acids, in both DPsP-B and WPsP-A. DPsP-B primarily exhibited β-type glycosidic linkages, while WPsP-A predominantly displayed α-type glycosidic linkages, with a smaller fraction being β-type. These findings indicated differences in monosaccharide composition and structure between PSPs extracted using different methods. Overall, this study provided experimental evidence for future research on the structure–function relationship of PSPs.

## 1. Introduction

*Polygonatum sibiricum*, commonly known as Huangjing, refers to the dried rhizome of a perennial herbaceous plant from the Liliaceae family. It is a medicinal herb with a sweet taste and neutral properties, widely used in both traditional medicine and culinary practices [1]. *Polygonatum sibiricum* is rich in polysaccharides, flavonoids, saponins, lignans, and other beneficial functional components for the human body [2,3]. It is known for its spleen-tonifying, kidney-nourishing, lung-moistening, Qi-replenishing, and Yin-nourishing effects [4,5]. Among its active constituents, *Polygonatum sibiricum* polysaccharides (PSPs) play a significant role. PSPs exhibit antioxidant properties [6], regulate glucose and lipid metabolism [7], modulate the immune system [8], and possess anti-aging properties [9]. These diverse biological activities make PSPs highly valuable for numerous applications.

The neutral polysaccharide extracted from the hot water of crude PS.A predominantly comprises highly branched galactomannans and homogalactans, both of which exhibit notable biological activity [10]. Analysis of the monosaccharide composition and structure of PS indicates that galactose and rhamnose are the primary constituents. These components significantly enhance the proliferation of mouse T and B lymphocytes induced by cyclophosphamide and boost phagocytosis in peritoneal macrophages [11]. PS is rich in mucilage, which, when extracted, breaks down into glucose, fructose, and other monosaccharides [12]. The dried and processed rhizomes of *P. cyrtonema* yield purified polysaccharides (specifically fructans and galactans), which are inherently branched. These polysaccharides are noteworthy for promoting the growth of bifidobacteria and lactic acid bacteria, showcasing their prebiotic potential [13]. Huangjing’s polysaccharides, used frequently in traditional medicine, exhibit varied biological activities after multiple steaming and drying treatments. Notably, the molar ratio of monosaccharide undergoes a transformation. After being subjected to four steaming procedures, there’s a significant uptick in its molecular weight, and the surface morphology becomes more compact [14]. From Huangjing, a novel branched polysaccharide (PSPW-3-A) is isolated using graded ethanol precipitation and column chromatography. With a molecular weight of 1.29 × 10^4^ Da, this polysaccharide demonstrates impressive immunoregulatory properties [15].

Deep eutectic solvents (DESs) are a novel category of environmentally friendly solvents, which consist of a hydrogen bond donor (HBD) and a hydrogen bond acceptor (HBA) with a certain molar ratio [16]. They possess weak acidity, are cost-effective and readily accessible, and demonstrate excellent biodegradability, stability, and low to no toxicity [17]. DESs facilitate the degradation of lignin and cellulose present in plant cell walls, enabling the extraction of bioactive compounds from plants [18]. They have found extensive application in the extraction of various active constituents from plants, including polyphenols, flavonoids, and polysaccharides. An ultrasound-assisted DES extraction approach was developed to extract polyphenolic compounds from *L. robustum*. Compared with traditional approaches, the new method has obtained more total phenolic and total flavonoid content and saved more extraction time, and the extraction showed potential biological activity [19]. Ternary deep eutectic solvents were also used to extract two flavonoids from *Ginkgo Biloba*, and the amount of quercetin and myricetin extracted was significantly increased [20]. A natural deep eutectic solvent was used to extract the polysaccharides from abalone viscera, to enhance extraction efficiency and obtain more glucuronic acid and stronger antioxidant capacity [21]. In addition, polysaccharides extracted using DES have the advantages of high yield, low impurities, and high antioxidant activity, and, therefore, have received more attention. Hot-water extraction (HWE), also referred to as decoction, is a technique used to extract medicinal substances through boiling them in water to obtain an extract. The prolonged exposure to high temperatures helps in softening and breaking down the plant cell walls, facilitating the release of polysaccharides [22].

In previous studies, our research team successfully optimized the extraction process of PSPs using DESs, resulting in a significant increase in polysaccharide yield of (33.81 ± 0.36)%, which was approximately four times that of the HWE method. In addition, the in vitro antioxidant capacity of polysaccharides extracted using DESs was significantly better than that of polysaccharides extracted using HWE. The results of this research have been published. Building upon this, the present experiment aimed to conduct a comparative analysis of the basic structural characteristics of PSPs extracted through the HWE and DES methods, focusing on molecular weight, molecular weight distribution, monosaccharide composition, functional groups, and glycosidic linkage types. Through the purification process, we obtained valuable insights that offer experimental evidence for the selection and refinement of polysaccharide extraction methods.

## 2. Results and Analysis

### 2.1. Molecular Weight and Molecular-Weight Distribution

During the analysis of polysaccharides using HPGPC, the appearance of a single, symmetrical peak in the chromatogram is indicative of a uniform polysaccharide [23]. The dispersity coefficient (D) is calculated as the ratio of weight-average molecular weight (Mw) to number-average molecular weight (Mn) and is used to assess the uniformity of the polysaccharide. A D value closer to 1 indicates a narrower range and a more uniform molecular-weight distribution [24,25]. Table 1 presents the results for molecular weight and molecular-weight distribution of PSPs extracted using two different methods. It is evident that among the three components of DPsP, DPsP-A and DPsP-C display a single peak with retention times of 33.644 min and 43.651 min, respectively. This finding indicated that DPsP-A had a significantly larger molecular weight compared with DPsP-C. The Mw and Mn of DPsP-A were 699,031 Da and 403,877 Da, respectively. However, for DPsP-C, these values were 7484 Da and 6151 Da, respectively. The dispersity indices of DPsP-A and DPsP-C were 1.73 and 1.22, respectively, suggesting that both polysaccharide components exhibited a certain level of uniformity, but DPsP-C demonstrated a slightly higher degree of uniformity with a narrower molecular-weight distribution. On the contrary, DPsP-B exhibited two absorption peaks with retention times of 39.873 min and 43.474 min. The Mw and Mn of one peak were 41,496 Da and 29,858 Da, respectively. For the other peak, they were 8109 Da and 6624 Da, respectively. The corresponding dispersity indices were 1.39 and 1.22.

From Table 1, it is apparent that, among the three components of WPsP, both WPsP-A and WPsP-C exhibited a single peak, with retention times of 43.664 min and 43.819 min, respectively. The Mw and Mn of WPsP-A were 7440 Da and 6118 Da, respectively. For WPsP-C, these values became 6935 Da and 5734 Da, respectively. The dispersity coefficients were 1.22 and 1.21, respectively, indicating that both WPsP-A and WPsP-C demonstrated good uniformity with similar levels of molecular-weight distribution. Conversely, WPsP-B exhibited two peaks, with retention times of 40.023 min and 43.819 min. The Mw and Mn of one peak were 38,768 Da and 28,043 Da, respectively. For the other peak, these values became 7257 Da and 5979 Da, respectively. The corresponding dispersity indices were 1.38 and 1.21.

It is important to note that different extraction methods, or even the same extraction method, can lead to significant variations in the molecular weight and molecular-weight distribution of polysaccharide components [26,27]. The study by Liu et al. showed that the molecular weight of *Chlorella* sp. polysaccharides extracted using the alkali method was higher than that extracted using the hot-water method. The high temperature degraded the polysaccharides to the lower molecules and destroyed the carboxyl group in the polysaccharides [28]. HPGPC analysis provides an approximate estimation of molecular weight and molecular-weight distribution. It relies on a calculation using glucan standard curves of varying molecular weights, and the results can be influenced by a choice of different types of standard samples [29].

### 2.2. Monosaccharide Composition Analysis

The monosaccharide composition of polysaccharides plays a vital role in analyzing their physicochemical and structural properties. Polysaccharides extracted through various methods have been reported to comprise neutral or acidic polysaccharides [30,31]. The analysis of monosaccharide composition can be conducted using HPLC and GC-MS methods [32,33]. In this study, the monosaccharide compositions and their respective proportions of PSPs extracted using the two extraction methods were determined using GC-MS. The results are illustrated in Table 2.

From Table 2, it is evident that DPsP-A and DPsP-B consisted of four monosaccharides: arabinose, mannose, glucose, and galactose, with monosaccharide ratios of 0.069:0.660:0.117:0.153 and 0.060:0.491:0.406:0.043, respectively. DPsP-C was composed of mannose and glucose, with a ratio of 0.559:0.441. WPsP-A and WPsP-B comprised three monosaccharides: mannose, glucose, and galactose, with monosaccharide ratios of 0.730:0.233:0.037 and 0.566:0.417:0.017, respectively. WPsP-C consisted of mannose and glucose, with a ratio of 0.418:0.582. These results indicated variations in the monosaccharide compositions of polysaccharides extracted using the two different approaches. The main components were mannose and glucose, while the polysaccharide fraction extracted using DESs also contained arabinose. Different extraction methods can influence the physicochemical properties of polysaccharides, including their chemical and monosaccharide compositions. It has been reported that *Polygonatum sibiricum* is rich in fructose, but no fructose was detected in this study. This could be due to the degradation and aggregation effects of polysaccharides caused by high-temperature treatment during HWE or the use of DESs, resulting in hydrolysis into glucose and mannose. Changes in monosaccharide composition may be attributed to the thermal treatment or to solvent-induced degradation, leading to the cleavage of glycosidic bonds [34,35]. Previous studies have shown that the main monosaccharides of grape seed polysaccharides extracted using DESs include galactose, arabinose, mannose, and glucose [36]. *Chlorella* polysaccharides extracted via the hot-water method contain the same monosaccharides, including rhamnose, arabinose, xylose, mannose, glucose, and galactose in different proportions [28]. Therefore, polysaccharides extracted using various methods exhibit a certain degree of similarity in their monosaccharide compositions. However, notable variations exist in the proportions among their constituents.

### 2.3. Infrared Spectroscopy

**Figure 1:**
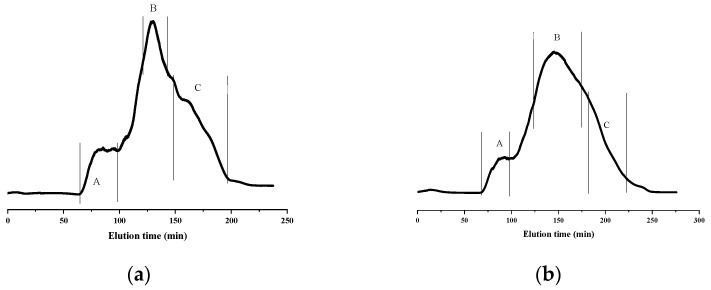
Elution curve of DPsP and WPsP based on superdex-200 column. (**a**) DPsP; (**b**) WPsP.

FTIR spectroscopy is a valuable tool for analyzing the structure of polysaccharides. It offers insights into the connectivity of bonds, functional groups, and other compositional features through examining the shape, position, and intensity of peaks in the spectrum [37]. In this study, the structural properties of purified DPsP and WPsP were assessed using infrared spectroscopy, and the corresponding findings are illustrated in Figure 1.

All constituents of DPsP and WPsP exhibited a weak absorption peak within the range of 4000–3600 cm^−1^, indicating the presence of free hydroxyl groups in the samples. The broad and smooth absorption peaks at 3444.24 and 3384.45 cm^−1^ were attributed to the stretching vibrations of hydroxyl groups within or between polysaccharide molecules. The absorption peaks at 2937.05 and 2927.41 cm^−1^ corresponded to the stretching vibrations of -CH_2_ groups. Additionally, the weak absorption peaks at 2376.87 and 2364.30 cm^−1^ could be attributed to the presence of CO_2_ in the surrounding air. The peaks around 1654.62 and 1658.48 cm^−1^ were attributed to the symmetric and asymmetric stretching vibrations of -COO- groups, indicating that both DPsP and WPsP extracted from *Polygonatum sibiricum* were acidic polysaccharides. DES is widely recognized as an efficient solvent for extracting polysaccharides from plants. Deep eutectic solvents and water were used to extract polysaccharides from *wolfberry*, and the yield of polysaccharides extracted using DESs was higher than the yield of those extracted using water. Compared with the water extraction approach, the polysaccharides extracted using DES exhibited higher total neutral sugar and uronic acid contents as well as lower molecular weight [38].

**Figure 2:**
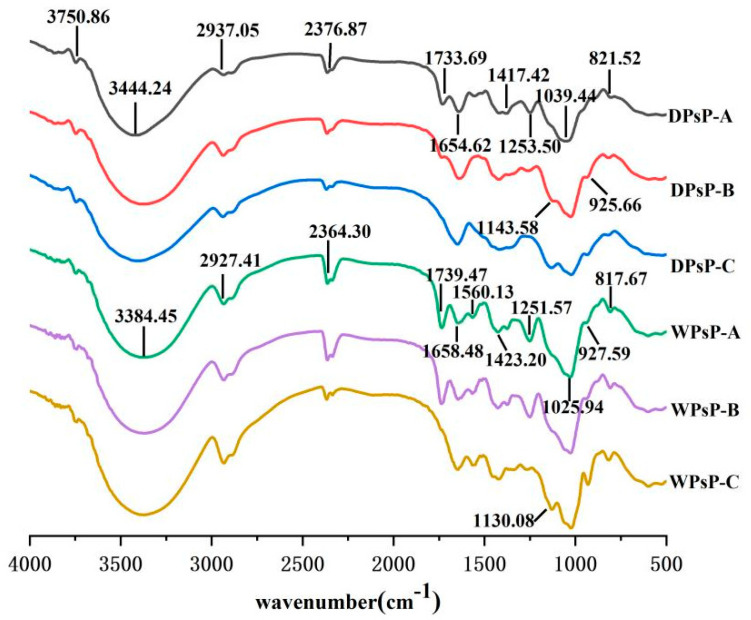
FTIR spectrum of polysaccharides from *Polygonatum sibiricum.*

The region below 1300 cm^−1^ was the fingerprint region of the infrared spectrum, where peaks were attributed to molecular structures and vibrations. The absorption peaks at 1039.44 and 1025.94 cm^−1^ were characteristic peaks of pyranose sugars, indicating the presence of pyranose sugars in the samples. The absorption peaks at 821.52 and 817.61 cm^−1^ indicated the presence of α-glycosidic linkages. Furthermore, variations could be observed in the infrared spectra among the different components of DPsP and WPsP. For instance, DPsP-A, DPsP-B, WPsP-A, and WPsP-B exhibited absorption peaks near 1733.69 cm^−1^ and 1253.50 cm^−1^, indicating the presence of C=O carbonyl groups and free carboxyl groups. In contrast, DPsP-C and WPsP-C did not display these peaks. Additionally, the absorption peaks at 927.59 cm^−1^ in WPsP-A, WPsP-B, and WPsP-C, as well as at 925.66 cm^−1^ in DPsP-B and DPsP-C, suggested the presence of β-glycosidic linkages in these respective polysaccharide components (Figure 2).

### 2.4. NMR Analysis

#### 2.4.1. NMR Spectrum Analysis of DPsP-B

**Figure 3:**
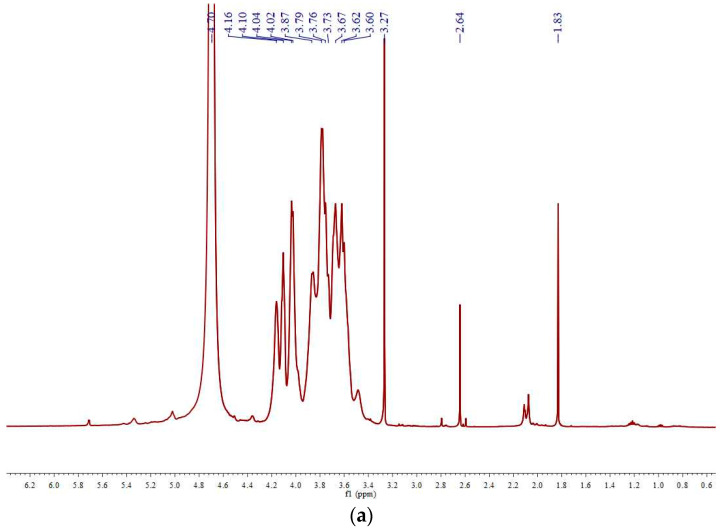

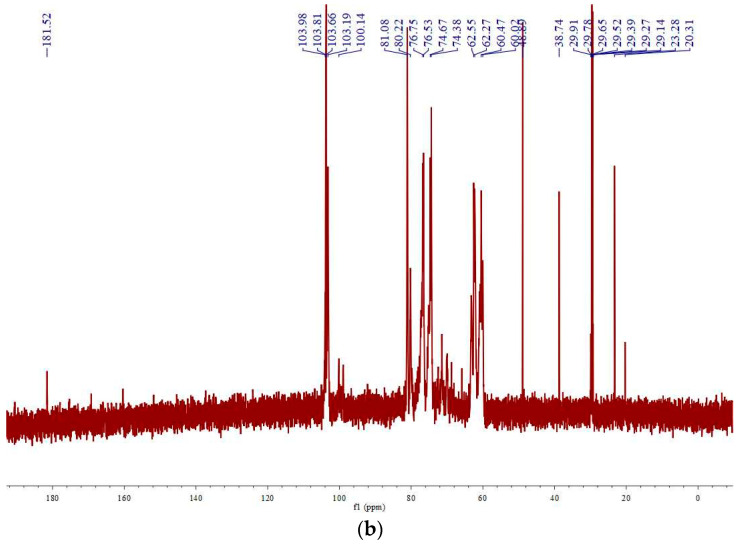
^1^H-NMR (**a**) and ^13^C-NMR (**b**) spectra of polysaccharides from *Polygonatum sibiricum* (DPsP-B).

The ^1^H-NMR and ^13^C-NMR spectra of DPsP-B are depicted in Figure 3a,b, respectively. In the ^1^H spectrum, the majority of peak positions were concentrated within the chemical shift range of 3.2–5.2 ppm, which are typical characteristic peaks of polysaccharides. The region spanning 3.2–4.3 ppm corresponds to proton signals from the sugar ring, while the range of 4.3–5.5 ppm corresponds to signals from the anomeric protons. Notably, peak positions above 5 ppm indicate the α-configuration of the glycosidic linkage, whereas positions below 5 ppm suggest the β-configuration [39].

The ^1^H-NMR spectrum of DPsP-B revealed several peaks below 5 ppm, indicating that the glycosidic linkages in DPsP-B predominantly adopted the β-configuration. Additionally, there were weak peaks above 5 ppm, suggesting the presence of α-glycosidic linkages, which was consistent with the findings from infrared spectroscopy. The peak at 1.83 ppm corresponded to the methyl protons, and the peak at 4.70 ppm was attributed to the solvent used in the measurement.

^13^C-NMR spectroscopy is commonly used to analyze the configuration of anomeric carbons and the substitution positions of polysaccharide residues. The chemical shift range of 90–110 ppm corresponds to the signals of anomeric carbons. In the case of DPsP-B, there were five peaks in the anomeric carbon region, specifically at 103.98, 103.81, 103.66, 103.19, and 100.14 ppm, suggesting the presence of five different sugar residues in DPsP-B. Among them, the chemical shifts of four sugar residue peaks were >103 ppm, indicating a predominance of β-configuration in the anomeric carbons of DPsP-B. The ^13^C-NMR spectrum of DPsP-B displayed no peaks at 82–84 ppm, but it did exhibit peaks at 62–63 ppm, suggesting the presence of pyranose sugar structures and the inclusion of a galactose residue. Notably, there was no peak observed at 18 ppm, indicating the absence of fucose, which was in line with the results obtained from infrared spectroscopy and monosaccharide composition analysis. Furthermore, the signal peak at 181.52 ppm suggested the presence of uronic acids in DPsP-B.

#### 2.4.2. The NMR Spectra of WPsP-A

**Figure 4:**
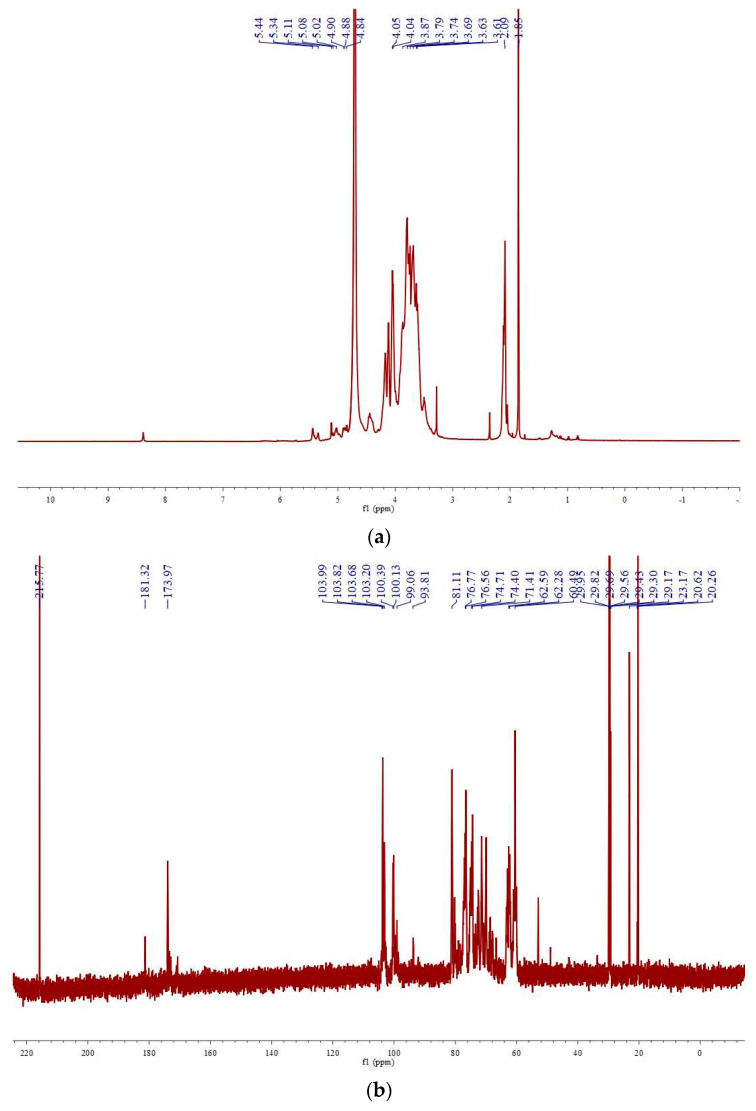
^1^H-NMR (**a**) and ^13^C-NMR (**b**) spectra of polysaccharides from *Polygonatum sibiricum* (WPsP-A).

The ^1^H-NMR and ^13^C-NMR spectra of WPsP-A are shown in Figure 4, panels a and b, respectively. As indicated by the ^1^H-NMR spectrum, the signal peaks of WPsP-A were concentrated between 3.5 and 5.5 ppm, which are typical for polysaccharides. The presence of signal peaks around 5 ppm in the ^1^H-NMR spectrum suggested that WPsP-A contained a comparable number of α and β glycosidic linkages.

The ^13^C-NMR spectrum of WPsP-A exhibited eight signal peaks within the anomeric carbon region, indicating the potential presence of eight sugar residues in the polysaccharide. The absence of signal peaks at 82–84 ppm suggested the presence of pyranose sugars in WPsP-A, while the signal peaks at 62–63 ppm suggested the inclusion of galactose residues, which was consistent with the monosaccharide composition analysis. Additionally, the signal peaks at 173.97 and 181.32 ppm corresponded to signals from uronic acid residues. It is important to note that polysaccharides have large molecular weights and complex structures, often resulting in significant peak overlap in one-dimensional spectra. Therefore, a more comprehensive analysis and investigation utilizing two-dimensional NMR detection is necessary [40].

## 3. Materials and Methods

### 3.1. Materials

*Polygonatum cyrtonema Hua* (*Pc H*) was sourced from the Wanbao Mountain Base of Lvyuan Agricultural and Forestry Technology Co., Ltd., located in Xinhua County, Loudi City, Hunan Province, China. Fresh rhizomes of *Pc H* were thoroughly washed, sliced, and subjected to hot air drying at 60 °C until the moisture content dropped below 10%. Subsequently, the dried rhizomes were finely ground into a powder using a cryogenic low-temperature grinder and stored in a sealed container at low temperature for future utilization.

### 3.2. Reagents

Choline chloride, urea, thiourea, citric acid, oxalic acid, sulfuric acid, anthrone, and other reagents were of analytical grade and purchased from Shanghai Pharmaceutical Group Chemical Reagent Co., Ltd., Shanghai, China. Dextran, fucose, arabinose, rhamnose, xylose, mannose, and other standard substances were obtained from Sigma-Aldrich, St. Louis, MO, USA.

### 3.3. Extraction of PSPs Using DES and HWE Methods

Deep eutectic solvents consisting of choline chloride(HBA) and 1,4-butanediol(HBD) with a molar ratio of 1:4 were used, and the mixture was stirred at 60 °C until a homogeneous colorless liquid was formed [34]. Next, 10 g of *P. cyrtonema Hua* powder was accurately weighed and placed in a conical flask, and an appropriate amount of DES was added with a liquid-to-solid ratio of 1:40. The extraction of PSPs was performed in a thermostatted water bath with a time of 48 min and a temperature of 89 °C. After cooling, the mixture was mixed with four volumes of anhydrous ethanol and placed in a refrigerator at 4 °C for 12 h. The precipitate was collected via centrifugation (5000 rpm, 10 min), dissolved in a certain volume of distilled water, and filtered to obtain the aqueous solution of PSPs. The Sevage reagent (chloroform: n-butanol = 4:1, *v*/*v*) was used to remove proteins, followed by concentration and ethanol precipitation for 6 h. The precipitate was centrifuged and freeze-dried to obtain DPsP. In parallel, the HWE method was used to extract PSPs (referred to as WPsP) following the procedures specified in the Chinese Pharmacopoeia 2015 edition [41].

### 3.4. Separation and Purification of PSPs

The Superdex-200 dextran gel purification system, coupled with a refractive index detector, was employed for the separation and purification of PSPs (DPsP and WPsP) [42]. In brief, 100 mg of DPsP or WPsP was accurately weighed and transferred to small beakers. Subsequently, 3 mL of distilled water was added for dissolution. The resulting solution was then transferred to centrifuge tubes and subjected to centrifugation at 12,000 rpm for 10 min. The supernatant was carefully collected and loaded onto the Superdex-200 dextran gel purification system. The fractions were collected based on the chromatogram (Figure 1). The collected polysaccharide solutions were subjected to vacuum-concentration and freeze-drying processes to obtain purified PSPs. Following the separation and purification steps, the polysaccharides obtained from DPsP were designated as DPsP-A, DPsP-B, and DPsP-C, while those obtained from WPsP were designated as WPsP-A, WPsP-B, and WPsP-C.

### 3.5. Determination of Molecular Weight and Molecular-Weight Distribution

The molecular weight of each component of DPsP and WPsP was determined using high-performance gel permeation chromatography (HPGPC) [43,44]. Glucose polymers of different molecular weights (Mw 1152, 11,600, 23,800, 48,600, 80,900, 148,000, 273,000, and 409,800 Da) were used as standards. Precise amounts of samples and standards were weighed and prepared as 5.0 mg/mL solutions. The solutions were centrifuged (12,000 rpm, 10 min), and the supernatant was filtered through a 0.22-μm microfiltration membrane. Then, the samples were transferred to 1.8-mL vials, and an injection volume of 20 μL was used.

### 3.6. Determination of Monosaccharide Composition

The monosaccharide composition analysis of PSPs was conducted using GC-MS [45]. Each component of DPsP and WPsP (20.0 mg) was taken and mixed with 1.0 mL of 2 mol/L trifluoroacetic acid for hydrolysis at 120 °C for 90 min. The hydrolysis products were washed with methanol 2–3 times and concentrated under a vacuum until no liquid remained. Acetylation of the products was then performed. The residue was dissolved in 2.0 mL of double-distilled water, followed by the addition of 100 mg of sodium borohydride for reduction, and allowed to react for 8 h. After neutralization with ice acetic acid, the sample was dried in an oven at 110 °C. Subsequently, 1.0 mL of acetic anhydride was added, and the reaction was carried out at 100 °C for 1 h. After cooling to room temperature, 3.0 mL of methylene chloride was added, and the solution was vacuum-concentrated and dried. This process was repeated 4–5 times to remove excess acetic anhydride. The resulting acetylated polysaccharide product was dissolved in 3.0 mL of chloroform and transferred to a separating funnel. A small amount of distilled water was added and thoroughly shaken, and the upper aqueous layer was discarded. This step was repeated 4–5 times. The chloroform layer was dried with anhydrous sodium sulfate to a volume of 10 mL and subjected to GC-MS analysis. The GC-MS conditions for monosaccharide composition determination were as follows. Column: RXI-5 SIL MS column (30 × 0.25 × 0.25). Temperature program: The initial temperature was set at 120 °C and then ramped up at a rate of 3 °C/min until reaching 250 °C. It was held at 250 °C for 5 min. Injector temperature: 250 °C. Detector temperature: 250 °C. Carrier gas: helium. Flow rate: 1.0 mL/min.

### 3.7. Infrared Spectroscopy

In brief, 1 mg of DPsP or WPsP components was individually mixed with 100 mg of potassium bromide in a mortar. The mixture was thoroughly ground and then pressed using a tablet press to produce uniform and transparent thin films. Fourier transform infrared spectroscopy (FTIR) scanning was conducted using an FTIR spectrometer [46]. The scanning range spanned from 4000 to 500 cm^−1^ with a scanning speed of 0.2 cm/s.

### 3.8. Nuclear Magnetic Resonance (NMR) Spectroscopy

WPsP-A and DPsP-B samples were analyzed and identified using 1D-NMR spectroscopy [47]. A sample amount of 0.05 g was utilized. The carbon spectrum resonance frequency was set at 150.9 MHz, and a 5 mm spinning tube with a spinning speed of 5 kHz was employed. The operating frequency for the experiment was 125.75 MHz, with a relaxation delay time of 20 ms, a contact time of 1 ms, and a pulse delay time of 3 ms. For the proton spectrum resonance, a frequency of 600.1 MHz was used, with a π/2 pulse length of 2.57 µs and a pulse delay time of 3 ms.

### 3.9. Data Processing and Analysis

Data analysis and plotting were carried out using Origin 8.5 software. For NMR data processing, MestReNova 6.1 software was utilized.

## 4. Conclusions

In conclusion, we compared the effects of DES extraction and HWE methods on the structure of DPsP and WPsP. The results of molecular weight and molecular weight distribution analysis showed that the molecular weights of DPsP components were slightly larger than those of WPsP, with DPsP-A exhibiting a slightly higher dispersity index and broader molecular weight distribution.

Both extraction methods resulted in polysaccharides with mannose and glucose as the predominant monosaccharide components. However, DPsP exhibited a slightly greater diversity of sugar components compared with WPsP.

The FTIR analysis indicated that all six components of PSPs exhibited characteristic absorption peaks associated with polysaccharides, with the majority being acidic pyranose sugars. The NMR results revealed that DPsP-B and WPsP-A contained pyranose sugars, along with galactose and uronic acid. In DPsP-B, the predominant glycosidic linkage was of the β-type, while in WPsP-A, the primary glycosidic linkage was of the α-type, with a minor fraction being of the β-type. These findings indicated that polysaccharides extracted using different methods exhibited variations in monosaccharide composition and structural characteristics.

## Figures and Tables

**Table 1:** Molecular weight data analysis of polysaccharides from *Polygonatum sibiricum.*

Samples	Retention Time (min)	Mw (Da)	Mn (Da)	D (Mw/Mn)
DPsP-A	33.644	699,031 ± 16,330 ^a^	403,877 ± 11,215 ^b^	1.73
DPsP-B	39.873 43.474	41,496 ± 2084 ^b^ 8109 ± 324 ^b^	29,858 ± 1049 ^a^ 6624 ± 256 ^b^	1.39 1.22
DPsP-C	43.651	7484 ± 319 ^a^	6151 ± 223 ^a^	1.22
WPsP-A	43.664	7440 ± 261 ^b^	6118 ± 299 ^a^	1.22
WPsP-B	40.023 43.719	38,768 ± 2108 ^b^ 7257 ± 291 ^a^	28,043 ± 1472 ^a^ 5979 ± 227 ^b^	1.38 1.21
WPsP-C	43.819	6935 ± 209 ^a^	5734 ± 178 ^a^	1.21

Data represented averages ± standard deviation. Significant differences (*p* < 0.5) were represented by different letters in each column.

**Table 2:** GC-MS data analysis of polysaccharides from *Polygonatum sibiricum.*

Monosaccharides Standards	Retention Time (min)	DPsP-A	DPsP-B	DPsP-C	WPsP-A	WPsP-B	WPsP-C
Rhamnose	20.767	-	-	-	-	-	-
Fucose	21.233	-	-	-	-	-	-
Arabinose	21.492	0.069 ± 0.002 ^b^	0.060 ± 0.003 ^a^	-	-	-	-
Xylose	22.808	-	-	-	-	-	-
Mannose	31.875	0.660 ± 0.010 ^a^	0.491 ± 0.006 ^a^	0.559 ± 0.005 ^c^	0.730 ± 0.012 ^c^	0.566 ± 0.005 ^c^	0.418 ± 0.007 ^b^
Glucose	32.142	0.117 ± 0.005 ^a^	0.406 ± 0.008 ^c^	0.441 ± 0.003 ^a^	0.233 ± 0.006 ^b^	0.417 ± 0.006 ^b^	0.582 ± 0.007 ^c^
Galactose	32.900	0.153 ± 0.007 ^a^	0.043 ± 0.002 ^b^	-	0.037 ± 0.006 ^a^	0.017 ± 0.005 ^a^	-

Data represented averages ± standard deviation. Significant differences (*p* < 0.5) were represented by different letters in each column.

## Data Availability

The data supporting reported results can be found at: Research on the extraction process and characteristics of Polygonatum polysaccharide deep eutectic solvent and its application.

## Sample Availability

Not available.
